# Diseases of the Heart and Circulation

**Published:** 1904-08-13

**Authors:** 


### DISEASES OF THE HEART AND CIRCULATION.

*(Continued from page 331.)*

**Diuretics.**[8]—In cardiac cases with dropsy, and in arteriosclerosis, theobromine is of great value, both in removing dropsy and as a hypnotic. The latter action is probably not due to any direct action but to clearing out of the system poisonous metabolic products producing sleeplessness. The drug may be given in cachets containing 7½ grains three times a day. A useful mixture is equal parts of theobromine, carbonate of lithium, and benzoate of sodium; or caffein may be given together with the theobromine. Theobromine has the disadvantage of dissolving with difficulty, and to meet this objection a soluble salt, called agurin, has been prepared. It is a combination of theobromine-sodium and acetate of sodium. Agurin can be given to the amount of 45 grains per diem in 7½ grain doses. Its diuretic action reaches a maximum on the third or fifth day after commencing administration, and persists for several days after its discontinuance. The greatest effect is produced in cases of arteriosclerosis of cardiac or renal origin. The diuretic effect is less pronounced in cases of ascites due to cirrhosis. The drug is nearly always well tolerated. A new diuretic, theocin, is reported on by Streit and by Stein. It is a substance prepared synthetically which is isomeric with theobromine. Caffein is, chemically, trimethylxanthin, whilst theobromine, both of whose double salts, diuretin and agurin, are serviceable diuretics, is dimethylxanthin. Theocin is a soluble substance, odourless and tasteless. Unlike caffein, it has no action on the heart-muscle and pulse. It may be given to the amount of 4 grains three times a day. Its diuretic effect is manifested in the first 24 hours, lasts about four days, and persists for two or three days after discontinuing the remedy. This diuretic effect is always obtained in oliguria of cardiac origin, and usually in chronic renal affections, but is generally absent in acute nephritis. Its action in pleural and peritoneal effusions is variable. To avoid unpleasant gastric disturbances the drug should be given in solution and after meals. Stein also reports excellent results in cardiac cases. In two of these digitalis had been previously given without success, and in another a combination of digitalis and diuretin.

**Caisson Disease and Diver's Palsy** are essentially the same disease, and due to the effects of too rapid effects of decompression. The men never suffer when under pressure, but only when they are brought rapidly to the normal atmospheric pressure. The recent experiments of Hill and Macleod[9] confirm those of Bert, who showed that the true cause of the affection is the effervescence of gas into the blood and tissue juices on decompression. Thus an animal exposed for four hours to eight atmospheres' pressure and quickly decompressed is like an opened bottle of soda-water. The fluids of the body generally effervesce. The varying symptoms of the disease are due to the varying seat of the air emboli. Young men escape the disease owing to the elasticity of their tissues and the greater facility for collateral pathways of circulation. The rules they lay down for this work are that the men should be between 20 and 25 years old, small and spare habit, and total abstainers. The longer the shift the greater is the saturation of the body fluids with gas, and the slower, therefore, should be the decompression. Similarly, the higher the pressure the shorter should be the shift and the longer the decompression. Thus, at two atmospheres' pressure, the shift may be 4 hours and the decompression ½ hour, whilst at five atmospheres' the shift should only be 1 hour and the time of decompression 1 to 2 hours.

**Aneurysm of the Abdominal Aorta.**—The cases which have occurred at Guy's Hospital during a period of 46 years have been collected and analysed by Bryant.[10] In this long period only 54 occurred, and they formed 16 per cent. of the total number of cases of aneurysm of the aorta; 77 per cent. of the number affected persons between 21 and 50 years of age, and 90 per cent. were in males. The two main factors in its causation are syphilis and violent exertion. The saccular and diffuse forms are by far the commonest, and the latter was usually the result of the rupture of a saccular aneurysm into the adjacent tissues. They most frequently spring from the neighbourhood of the cœliac axis. Erosion of the bodies of the vertebræ is one of the chief effects of the gradual increase in size of the sac. If death occurs as a direct result of the aneurysm, rupture is almost always found as cause; it is generally either into the retroperitoneal tissues or the peritoneal cavity. Of the symptoms pain is the most constant and usually the first indication, usually referred to the back or abdomen. If this is complained of and a deep-seated abdominal tumour with marked systolic bruit and expansile pulsation be found, the presence of an aneurysm may be assumed; but there must be no question about the expansile character of the pulsation. The condition which most frequently leads to mistake is pulsating abdominal aorta, but in this complaint the patient is generally a woman, and one who is anæmic, dyspeptic, and neurotic. Further, there is no tumour and no murmur unless the stethoscope is pressed hard on the aorta. There may be pulsation in the epigastrium from other causes, viz. enlargement of the right ventricle, pulsating liver, and pulsating sarcomatous tumours. In reference to the first point it is exceedingly rare for aneurysm to be associated with hypertrophy of the heart. A pulsating liver is associated with enlargement and with signs of heart or lung disease. Tumours of the pylorus pulsate, but the pulsation is not expansile, and they may be found to move downwards on inspiration. Pressure of an aneurysm on the ureter may give rise to an erroneous diagnosis of renal calculus or perinephric abscess. The prognosis is nearly always bad, and treatment very unsatisfactory. Of the various methods recommended the combination of introduction of wire and electrolysis holds out the greatest hope of cure.

[8] Pract., Feb. [9] Jour of Hygiene, Oct. [10] Clin. Jour., Nov. 18 and 25.

